# The journey of CAR-T therapy in hematological malignancies

**DOI:** 10.1186/s12943-022-01663-0

**Published:** 2022-10-08

**Authors:** Junru Lu, Guan Jiang

**Affiliations:** 1 Department of Dermatology, Affiliated Hospital of Xuzhou Medical University, Xuzhou, Jiangsu China; 2 Xuzhou Medical University, Xuzhou, Jiangsu China

**Keywords:** CAR-T cell therapy, Hematological malignancies, Targeted therapy, Combinatorial therapy, Drug product

## Abstract

Chimeric antigen receptor T (CAR-T) cells therapy has revolutionized the treatment paradigms for hematological malignancies, with multi-line therapy-refractory patients achieving durable complete remissions (CR) and relatively high objective response rate (ORR). So far, many CAR-T products, such as Kymriah, Yescarta and Tecartus, have been developed and got the unprecedented results. However, some patients may relapse afterwards, driving intense investigations into promoting the development of novel strategies to overcome resistance and mechanisms of relapse. Notable technical progress, such as nanobodies and CRISPR-Case9, has also taken place to ensure CAR-T cell therapy fully satisfies its medical potential. In this review, we outline the basic principles for the development and manufacturing processes of CAR-T cell therapy, summarize the similarities and differences in efficacy of different products as well as their corresponding clinical results, and discuss CAR-T immunotherapy combined with other clinical effects of drug therapy.

**Supplementary Information:**

The online version contains supplementary material available at 10.1186/s12943-022-01663-0.

## Background

Cancer treatment approaches have rapidly developed in recent years and particularly, immunotherapy is one of the fastest developing cancer treatment options. CAR-T cell immunotherapy has achieved impressive results in cancer treatment and immunotherapy has gradually become the key treatment option for malignant hematological conditions [1]. From Kymriah and Yescarta, which were first to be approved by the FDA in August and October 2017 to treat leukemia and lymphoma, to the latest advances, such as CB-010 therapy, they all play a critical role in malignant tumors, particularly in cases of relapse and refractory cancers.

Although CAR-T cell immunotherapy may significantly improve clinical outcomes, there is a need to overcome the challenges associated with its safety and efficiency, especially the toxicities of CAR-T cell therapy after administration. Mortality and morbidity can occur due to two main complications—cytokine release syndrome (CRS) and immune effector cell-associated neurotoxicity syndrome (ICANS). Additionally, other serious adverse reactions may occur, including tumor lysis syndrome and off-tumor target toxicity. A great deal of labor and production cost and relapse after treatment are also major challenges in the treatment process.

While there are many issues, CAR-T cell immunotherapy is still a significant novel concept. More clinical trials have been registered to assess the clinical efficiency of CAR-T cell immunotherapy on hematological tumors. To enhance the therapeutic effects, numerous methods have been developed, including modulation of CAR-T cell activities [2]. CAR-T immunotherapy in combination with checkpoint blocking antibodies or small molecule inhibitors has appeared better clinical outcomes compared to single-agent approaches (NCT03331198 (TRANSCEND CLL 004), NCT03960840) (see Additional file 2: Table 2 for the details) [3, 4].

## Mechanisms and development of CAR-T cell immunotherapy

**Fig. 1:**
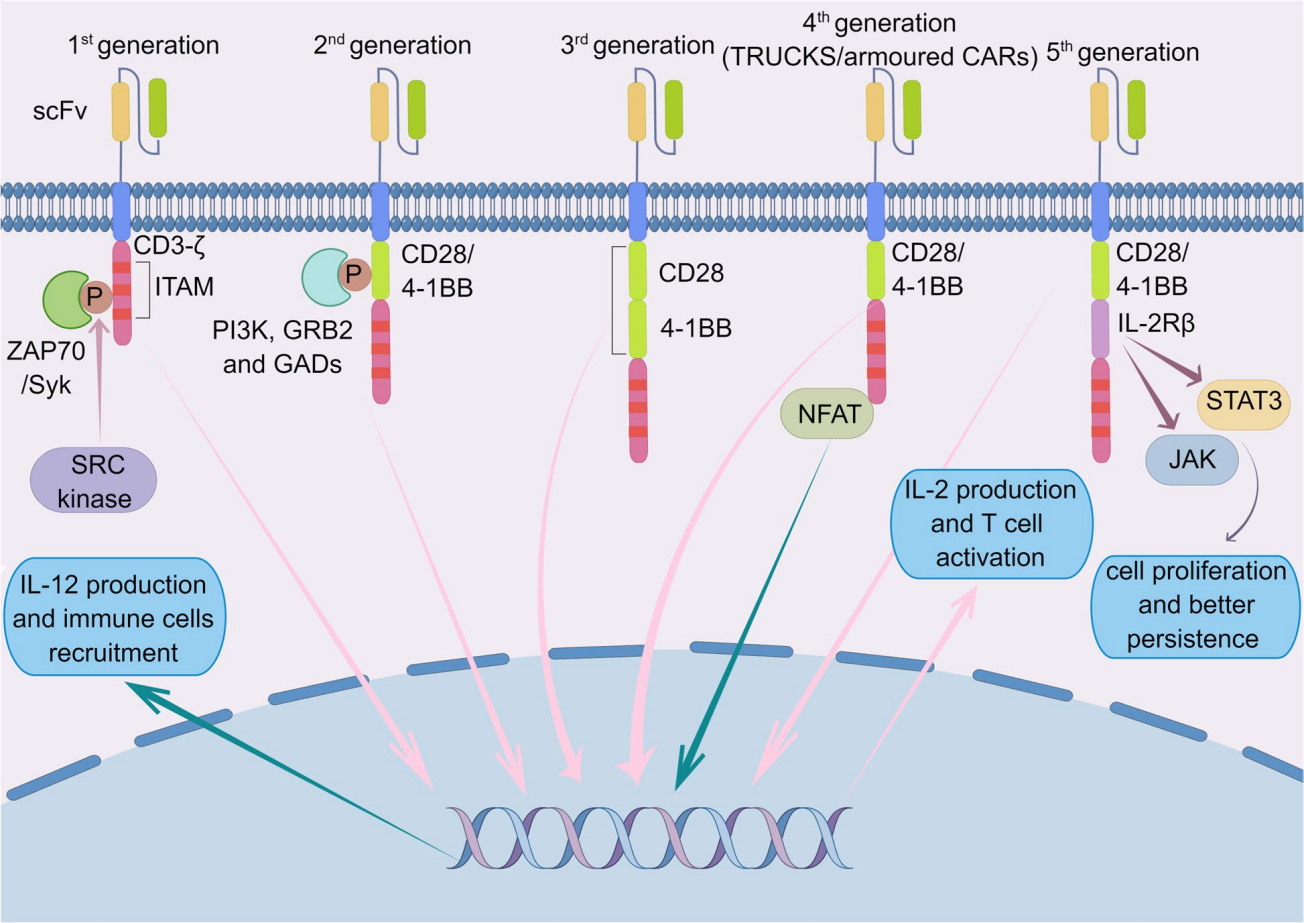
The development processes of five generations of CARs The first generation only had the intracellular CD3-ζ signal molecule, which are phosphorylated via SRC tyrosine kinase family. In the second generation, CD28 or 4-1BB co-stimulatory region was integrated with the CD3-ζ molecule. Various proteins containing SH2 domain (PI3K, GRB2 and GADs) are recruited while IL-2 is induced. Based on the second generation, the third generation (such as CAR 22–19) added two different co-stimulatory domains (CD28–4-1BB/ICOS-4-1BB). The fourth generation (TRUCKs or armoured CARs) paired with a constitutively expressed chemokine (IL-12). The fifth generation, also based on the second generation, added intracellular domains of cytokine receptor (IL-2Rβ). The activation of JAK-STAT, deriving from IL-2Rβ and incorporated between CD28/4-1BB and CD3-ζ, stimulates cell proliferation. (By Figdraw)

**Fig. 2:**
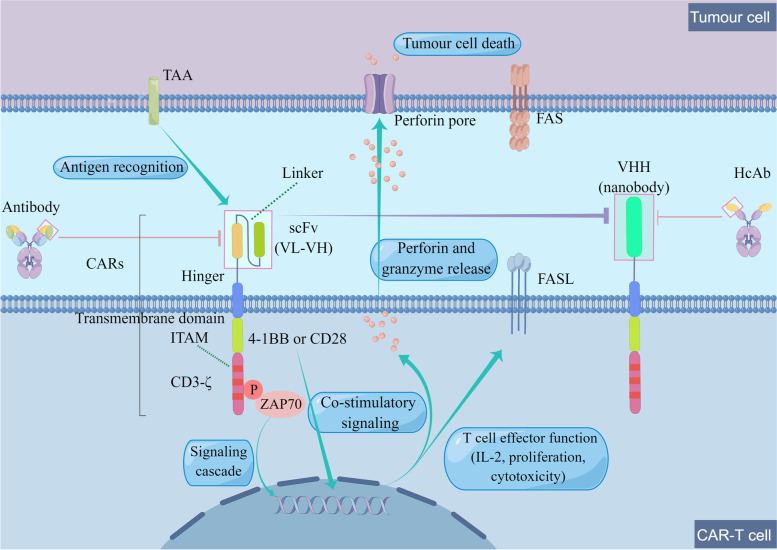
Mechanisms of CAR-T therapy A monoclonal antibody is usually used to generate the extracellular portion of the CARs. The scFv, able to recognize TAAs, is composed of the VL and VH region. In addition to scFv, the targeting domain also use VHH, also known as nanobodies, which are derived from the variable domain of HcAbs. The hinge region connects the transmembrane region and extracellular region. The intracellular activation region can be divided into co-stimulatory region and signaling region. Signals generated by co-stimulatory mechanisms depend on the co-stimulation domain (CD28 depends on PI3K, while 4-1BB employs NF-κB and TRAFs). CD3-ζ is usually a signal region with three ITAMs. Once the scFv recognizes and binds TAAs, Phosphorylation of the ITAMs initiates signal transduction through ZAP70, and then sends out signals to release T cell response (granzyme and perforin/BID or FADD). (By Figdraw)

Eshhar et al. were the first to design the CAR-T cell technology to express antigen receptors [5]. Up to now, there are five generations of CAR structures (CARs). Figures 1 and 2 show the development processes of five generations of CARs and mechanisms of CAR-T therapy, respectively.

### CAR parts

The CARs are made up of the extracellular antigen-binding, hinge transmembrane and intracellular activation region [6]. The intracellular activation region is composed of co-stimulatory and signaling regions. The variable heavy (VH) and variable light (VL) chains, also called the single-chain variable fragment (scFv), are joined by a repetition of glycine and serine residues, and a few Whitlow linker molecules [7]. The scFv recognizes specific tumor-associated antigens (TAAs) [8], including proteins, glycoproteins, and other elements while signaling regions principally enhance T-cell proliferation and differentiation. This principle is on a similar basis to antigen-antibody complementarity, which can bypass major histocompatibility complex (MHC) antigen presentation and allow TAAs to directly activate T cells. Therefore, CAR-T therapy can enable access to a large subset of patients. Additionally, single variable domain on a heavy chain (VHH), also called nanobodies, have also been employed as the targeting domain of CARs [9]. VHH are derived from the variable domain of heavy chain-only antibodies (HcAbs), produced by the Camelidae family and sharks naturally [10, 11]. Compared to conventional scFv, VHH recognize and bind target antigens with similar binding ability and specificity, as well as the solubility and stability [10]. Even if VL and constant domains are absent, VHH can still possess these characteristics [10]. Therefore, VHH-based targeting CAR-T cells could be as effective as traditional CAR-T cells with scFv-based targeting domains.

The hinge region (the spacer region) connects the extracellular region and the transmembrane region. The CARs design uses various hinge regions to achieve flexibility so that scFv domain can bind TAAs, including domains that develop from CD28 to CD8 [12]. Generally, the transmembrane moiety also derives from CD28 and CD8, which can affect molecular interactions between CARs by endogenous transmembrane binding of the original protein to form a homodimer or a trimer [13].

The main co-stimulatory regions are CD28 and 4-1BB, while the other common co-stimulatory regions include CD27, OX40, and ICOS [14–16]. CAR-T cells with different co-stimulatory regions have shown different dynamics. Compared with the presence of CD28, which resulted in rapid expansion but less durability, CARs with the presence of 4-1BB conferred slower expansion but longer persistence [17, 18]. Since T helper-2-associated cytokine levels were rapidly elevated, CD28-containing CAR-T cells exhibit a more effector-like memory phenotype and enhance glycolytic metabolism [19–21]. Therefore, the CD28 co-stimulatory pathway is rapidly activated in CAR-T cells, which may lead to early-onset CRS. 4-1BB-based T cells can enhance mitochondrial activities and become more central memory-like T cells with increased expiratory capacity and fatty acid metabolism [21, 22]. .4-1BB CARs can activate non-canonical nuclear factor-κB (NF-κB) signaling after ligand engagement and depend on tumour necrosis factor (TNF) receptor-associated factors (TRAFs) [23]. Through regulating NF-κB, TRAFs can affect the viability, expansion and cytotoxicity [24]. The difference between these two co-stimulation pathways should be viewed comprehensively, because variables may exist causing the persistence or failure of T cells and no clinical data compare both CAR head-to-head.

The CD3-ζ (CD247) is the most distal intracellular part and is a signal region with three immunoreceptor tyrosine-based activation motifs (ITAMs) [25]. Phosphorylation of the ITAMs initiates signal transduction via tyrosine kinase ζ-related protein of 70 kDa (ZAP70) [26], resulting in activated T cell responses (proliferation, cytokines release etc.).

### The development of CARs

Due to advances in related technologies, including genetic engineering and immunotherapy, CARs have experienced five generations. The original CAR only had intracellular a CD3-ζ signaling molecule [27]. The first generation of CARs was efficient at specifically recognizing tumor antigens and improving T cell anti-tumor activities. However its proliferative abilities are low while its therapeutic effects are minimal, because of the absence of co-stimulatory (such as CD27, CD28, CD134 and 4-1BB) and cytokine signaling, such as interleukin-2(IL-2) [28]. In second generation CAR, the CD28 or 4-1BB co-stimulatory region was integrated with the CD3-ζ molecule, and cell proliferation and cytotoxicity were significantly improved [29]. Using the second generation as a basis, the third generation (such as CAR 22–19 [30]) had two different co-stimulatory domains (CD28–4-1BB/ICOS-4-1BB) [31]. To enhance the ability to attack tumuor cells, the fourth generation of T cells redirect for universal cytokine killings (TRUCKs) or armoured CARs were developed, containing IL-12 expression system [32, 33]. By releasing important chemicals or cytokines in tumors, the fourth generation can improve tumor cytotoxicity by overcoming the immunosuppressive networks in the tomour microenvironment (TME) [8]. Moreover, TRUCKs can induce IL-12 activation downstream of the nuclear factor activated T cells (NFAT) transcription factor after CAR recognizes TAAs to recruit other immune cells (DC, phagocytes and NK among others) [34]. The fifth generation is based on the second generation, including truncated cytoplasmic IL-2 receptor β-chain (IL-2Rβ) domain and a STAT3-binding moiety [35]. Activating the fifth generation can promote T cell receptor (TCR) and cytokine-driven JAK-STAT signaling to enhance proliferation and activation of bioengineered T cells [35]. Although CARs evolve rapidly over the past three decades, it is still unknown which design of CARs can provide the best clinical efficacy because of no head-to-head studies performed.

### Mechanisms of CAR-T cell therapy

CAR-T cells mainly rely on engineered CARs, exhibiting antigen specificity and T-cell cytotoxicity [8]. The targets of hematological malignancies are concentrated on CD19, BCMA, CD20 and CD22. In addition, there are some other TAAs, CD23, CD30, CD33, SLAMF, ROR1, GRP78, CD138 etc. CD19, CD20, CD22, CD23 and ROR1 are usually used targets for B-cell malignancies [36–40]. Among them, CD19 is the most frequently used target and highly expressed in most B-cell malignancies [36]. CD30 is usually expressed on tumour cells of Hodgkin’s lymphoma [41]. CD33 is a favorable target for leukemia, especially acute myeloid leukemia (AML) [42]. SLAMF, BCMA and CD138 are developed to treat multiple myeloma (MM) [43–45], especially BCMA. When CAR-T cells recognize specific TAAs [8], ITAMs initiate activation via phosphorylation, promoting proliferation, releasing cytokines and boosting immune responses. However, this binding mechanism has certain limitations. Loss of expression of TAAs (such as CD19 or CD20) has been known as one of the key mechanisms of tumour resistance [46]. Furthermore, not the same as tumour specific antigens (TSAs), TAAs have low selectivity, which are overexpressed on tumours, but also expressed on normal organs and tissues [47], causing off-target effects and raising safety concern.

Cytotoxicity is usually exerted via; i. Cytokine secretion of granzyme and perforins by CAR-T cells [48], which is the primary way to inhibit tumour progression and ii. Stimulation of cancer cell apoptosis by activating the apoptosis signaling pathway, including activation of the BH3-interacting domain death agonist (BID) and FAS-associated death domain protein (FADD) [49, 50].

### Manufacturing engineering

CAR-T cells are derived from patients’ own cells (autologous). Therefore, they can avoid the problems of allogeneic rejection or graft-versus-host disease (GVHD), but they must be separately customized, which requires advanced technologies and financial resources. CAR-T cell manufacturing usually begins with leukapheresis to remove blood from the patient’s body and separate the leukocytes [51], while some researchers employ non-separate whole peripheral blood [52]. In the process of activation, a common approach is to add OKT3 (anti-CD3 monoclonal antibody) and IL-2. Excessive concentrations of IL-2 can cause dysfunction of T cells, poor effector function and quick apoptosis [53]. Furthermore, beads coated with anti-CD3/anti-CD28 monoclonal antibodies are also employed, which may retain memory phenotype more than with OKT3/IL-2 [54]. The overall T cell phenotype can also be manipulated using other cytokines, including IL-7 and IL-15, which may cause a higher percentage of memory subsets [55]. After activation, the CAR gene is usually introduced into the T cell genome via viral methods (lentiviruses and γ-retroviruses) [8], which is thought to be safe and effective. Of note, lentiviral transduction is predominant than retroviral transduction in current clinical trials [56]. In addition to viral methods, Cell electroporation by non-viral routes is a low-cost gene transfer approach whose safety and efficacy is yet to be evaluated [57]. During expansion, three approaches (plates/T-flasks, static culture bags and rocking motion bioreactors) can be employed [58–60]. Of note, the rocking motion bioreactor method for expansion is prevailing [56], utilizing the most advanced technology and suitable for large patient cohorts.

Of note, before transfusion, chemotherapeutic approaches (lymphodepleting conditioning regimen), such as the employment of antimetabolites (fludarabine and cyclophosphamide) for pretreatment should be used to eradicate regulatory T cells (Treg) and myeloid-derived suppressor cells (immunosupressive elements) [61]. Furthermore, the lymphodepleting conditioning regimen can enhance the efficacy of therapy by multiple mechanisms, including increase of homeostatic cytokines (IL-2, IL-7 and IL-15), induction of costimulatory molecules, downregulation of indoleamine 2,3-dioxygenase on the tumour, and promotion of potency of adoptively transferred T cells [61]. Moreover, chemotherapeutic drugs from different products have different administration times.

Even though CAR-T cell therapy has significant treatment effects, its long preparation cycle and complex process, as well as the need to tailor it for each patients are major limitations in its applications. Recently, a technology called Multifunctional Alginate Scaffold for T Cell Engineering and Release (MASTER) has been invented [62]. MASTER integrates T cell activation, reprogramming and in vivo amplification, reducing the time for manufacturing to 1 day. Of note, MASTER is modified with antibodies that activate T cells and are impregnated with ILs, thus, the cell activation process starts almost immediately. In mouse lymphoma xenotransplantation models, CAR-T cells generated in vivo entered the blood circulation and controlled the development of distant tumors, exhibiting stronger durability and better therapeutic effects than conventional CAR-T cells [62]. However, more further trials should be performed before this technique can be utilized in clinical applications. Additionally, another recent study has shown that quickly generated (within 24 hours) non-activated CAR-T cells exhibited higher anti-leukaemic in vivo activity per cell than activated CAR-T cells produced employing the standard protocol [63]. Compared to this innovative approaches, traditional manufacturing (activation and expansion) may cause progressive differentiation and the associated loss of anti-leukaemic activity, while this method may be more convenient and cheaper, also broadening their applicability.

## Clinical outcomes of CAR-T immunotherapy

In this section, the latest clinical findings on treatment of hematological malignancies using commercially available and state-of-the-art CAR-T products are summarized. Table 1 (See Supplementary Table 1, Additional file 1) shows the variations in safety and efficiency effects of different CAR-T products.

### Kymriah (Tisagenlecleucel)

Kymriah was the first approved CAR-T cell product (CD19/FMC63) by FDA on August 31, 2017 indicated, for the treatment of patients (below 25 years of age) with B-cell precursor acute lymphoblastic leukemia (B-ALL) that was in refractory or in second or later relapse [18]. On May 2, 2018, Kymriah was approved for the treatment of adult patients with relapsed/refractory large B-cell lymphoma (R/R LBCL) after two or more lines of systemic therapy, including diffuse large B-cell lymphoma (DLBCL), high grade B-cell lymphoma (HGBCL) and DLBCL arising from follicular lymphoma (FL) [64]. In JULIET (NCT02445248) phase 2 trial, 111 patients with R/R LBCL were infused. An ORR was 52%, while the CR rate was 40% [65]. The most common adverse events (AEs) are CRS and ICANS, incidences of which were 56% (22% ≥ Grade 3) and 21% (12% ≥ Grade 3), respectively [65]. In Additional file 1: Table 1, the incidence of CRS above Grade 3 was relatively high among comparable drugs.

On May 27, 2022, Kymriah was approved by FDA for a new indication for the treatment of adult patients with R/R FL after two or more lines of systemic therapy [66]. This approval is based on the global phase 2 ElARA trial (NCT03568461), marking the third indication for Kymriah and expanding the scope of diseases to be solved. In this trial, 97 patients (> 18 years of age), with R/R FL (Grade 1-3A) after ≥2 lines of therapy or failing autologous stem cell transplant, received Kymriah [67]. Of 94 patients evaluable for efficacy, the ORR was 86% and the CR was 66%. Any Grade CRS occurred in 49% of patients (Grade ≥ 3, 0%). Any Grade ICANS occurred in 9% of patients (Grade ≥ 3, 1%). These data demonstrate that Kymriah can be a promising therapy for patients with R/R FL, including high-risk patients after multiple lines of systemic therapy.

Of note, one patient with B-ALL, treated with CTL019 (Kymriah), relapsed and died 8 months after CR, which was attributed to the presence of incidental CD19 CAR-transduced B cells (CARB) in his bone marrow and circulatory system [68]. This report conferred resistance to subsequent CAR-T cell therapy, emphasizing the significance of a purified starting product. Moreover, during the process of leukapheresis, circulating tumour cells gathered together with lymphocytes can unintentionally be transduced with the CARs, which may cause antigen masking by binding of CARs expressed by tumour cells to TAAs on the same cells, leading to clonal expansion and tumour cell escape in vivo [68].

Combination therapy, including the combination of pembrolizumab or the Bruton’s tyrosine kinase (BTK) inhibitor, is also a preferred treatment option for aggressive lymphoma (described in detail below).

### Yescarta (Axicabtagene Ciloleucel)

Yescarta (Axicabtagene Ciloleucel, KTE-X19), the second generation of retroviral-transduced CD-28 based CAR-T cell product (CD19/FMC63), was approved by FDA on October 18, 2017 for the treatment of adult patients with R/R LBCL after two or more lines of systemic therapy, including DLBCL, primary mediastinal B-cell lymphoma (PMBCL), HGBCL and transformed follicular lymphoma (TFL) [69]. In a key Phase 1/2 study, ZUMA-1 (NCT02348216) [17], 101 patients received Yescarta infusion and showed ORR and CR of 83 and 58%, respectively [70]. Above 50% of treatment failures occurred within the first 3 months after Yescarta administration, while 75% of patients that responded within 3 months were still in remission without further interventions after the 2-year follow-up period [71]. CRS was detected in most of the patients (93, 11% ≥ Grade 3). ICANS was detected in 67% of patients (32% ≥ Grade 3).

Based on the results of ZUMA-5 (NCT03105336), the indication for adult patients with R/R FL after two or more lines of systemic therapy was approved by FDA on March 3, 2021. In this trial, 148 patients (≥18 years of age) with FL or marginal zone lymphoma (MZL) received an infusion of Yescarta [72]. Of 104 patients evaluable for efficacy, 96 patients (92%) had an ORR and 77 patients (74%) had a CR. Grade 3 or 4 CRS occurred in ten patients (7%) and Grade 3 or 4 ICANS occurred in 28 patients (19%). Yescarta demonstrated high rates of the ORR, and had a manageable safety profile in patients with R/R FL and MZL. On April 1, 2022, Yescarta was approved by FDA for the treatment of adult patients (≥18 years of age) with LBCL, who were R/R within 12 months of first-line chemoimmunotherapy (including anti-20 monoclonal antibody and anthracycline) and intended to proceed to high-dose therapy with autologous stem cell rescue. In the ZUMA-7 (NCT03391466) trial [73], the ORR and CR were 83 and 65%, respectively. Among 170 patients receiving Yescarta, any Grade CRS occurred in 92% of patients (Grade ≥ 3, 11%). Any Grade ICANS occurred in 60% of patients (Grade ≥ 3, 6%). Moreover, recent study (ZUMA-12) demonstrates safety and efficacy of Yescarta as a part of first-line treatment for adult patients with high-risk LBCL [74].

Under the trade name Yikaida (FKC876), Yescarta was approved in China in June 2021.

### Tecartus (Brexucabtagene Autoleucel)

Tecartus (Brexucabtagene Autoleucel) is the third CAR-T cell drug (CD19/FMC63) that was first approved for treating R/R mantle cell lymphoma (MCL) patients on July 24, 2020. A key Phase 2 clinical trial, ZUMA-2 (NCT02601313) [75] included all patients with MCL pretreated with BTK inhibitors who had ORR of 93% and CR of 67%. In Additional file 1: Table 1, the ORR of this drug is relatively high, however, the incidence of ICANS above Grade 3 reached 31%, ranking the first among its kind.

On October 1, 2021, FDA approved Tecartus for the treatment of adult patients 26 years of age and above with R/R B-ALL. In a phase 2 ZUMA-3 (NCT02614066) trial, 55 patients received infusion. Among them, CR or CR with incomplete hematologic recovery was observed in 39 patients (71%) [76]. CRS of any Grade occurred in 49 patients (89%) (24% ≥ Grade 3, 13 patients). ICANS of any grade occurred in 33 patients (60%) (25% ≥ Grade 3, 14 patients). Tecartus is a potential therapy, but further investigation is needed to decide where Tecartus will fit in current treatment methods or if Tecartus proves beneficial in other patients with ALL (T-cell ALL).

In terms of manufacturing, compared to the Yescarta, Tecartus involves T-cell selection and lymphocyte enrichment, which includes the removal of tumor cells cyclically expressing CD19 in the patient’s leukocyte isolate material. The T cells of MCL patients in the starting material are relatively small, whereas the numerous circulating tumor cells and leukemic blasts exist in the peripheral blood. Therefore, removing these cells may reduce the likelihood of production failure [77, 78].

Tecartus exhibited improvements in both ORR and CR, compared to its predecessor, Yescarta, but there was also an increase in incidences of CRS and ICANS above Grade 3. If the toxic effects of Tecartus can be controlled, it will be an ideal drug for treating hematological tumors.

### Breyanzi (Lisocabtagene Maraleucel)

On February 5, 2021, Breyanzi (Lisocabtagene Maralecel) (CD19/FMC63) was approved by FDA, indicated for treating adult patients with R/R LBCL after two or more lines of systemic therapy, including unspecified DLBCL (including TFL), HGBCL, PMBCL and 3B grade FL [79], Approval was based on results from TRANSCEND NHL 001 (NCT02631044), aimed at verifying the efficacy and safety of Breyanzi in R/R LBCL and MCL patients [80]. Among the 256 patients, an ORR was 73% and a CR was 53%, while the estimated 1-year duration of CR was 65% [81]. Any Grade CRS was 42% (≥Grade 3, 2%) and any Grade ICANS 30% (≥ Grade 3, 10%) [81].

On June 25, 2022, FDA approves Breyanzi for R/R LBCL after one prior therapy. Approval was based on results from the phase 2 PILOT trial (NCT03483103) and the pivotal phase 3 TRANSFORM trial (NCT03575351) [82, 83]. With this approval, Breyanzi now has the broadest patient eligibility of any CAR-T therapy in R/R LBCL. In TRANSFORM trial, 90 patients received infused. An ORR and a CR were 86% (79 patients) and 66% (61 patients), respectively. Any Grade CRS was reported in 49% of patients (only 1 patient had Grade 3 CRS). Any Grade ICANS was observed in 12 patients (≥Grade 3, 4%).

Compared to other CAR-T cell products, Breyanzi contains a non-functional truncated epidermal growth factor receptor (tEGFR) that is co-expressed with CD19-specific CAR on cell surfaces and can serve as a surrogate for CAR expressions [80]. During manufacturing process of Breyanzi, CD8^+^and CD4^+^T cells are selected from leukapheresis material and then independently activated, transduced, and expanded [84]. The manufacturing process leads to a clonally diverse, less differentiated pure T-cell product (memory T-cell) and CD19^+^cells below the level of quantitation [84]. Moreover, CD8^+^ and CD4^+^CAR-T cells are reinfused in a 1:1 ratio [81]. The use of a fixed transfer dose enhances the control of toxicities, thereby promoting persistence and amplification efficiency.

In Additional file 1: Table 1, Breyanzi had a higher safety and persistence profile, compared to the Grade 3 and above incidences of CRS and ICANS when other drugs were used.

### Abecma (Idecabtagene Vicleucel)

Abecma (Idecabtagene Vicleucel, bb2121) is the first CAR-T cell drug (BCMA/C11D5.3), approved by FDA on March 26, 2021. This product can be used to treat adult patients with R/R MM after fourth-line or more therapy, including immunomodulatory, proteasome inhibitors and anti-CD38 monoclonal antibodies [85]. BCMA (TNFRSF17 or CD269) is a transmembrane glycoprotein that belongs to the superfamily of TNF receptors and is naturally expressed on plasma cells [86]. In malignant plasma cells, its expressions are persistently abnormal, while in normal tissues, its expressions are very low and almost non-existent in CD34^+^hematopoietic stem cells. Since BCMA is selectively induced during plasma cell differentiation, it is virtually lacking in naive and memory B cells [86].

Due to poor clinical outcomes and low rates of achieving CR, a total of 140 patients (128 patients received infusion) diagnosed with R/R MM and receiving the above three treatments were involved in the KARMMA (NCT03361748) Phase 2 study [85]. About 73% of patients achieved ORR while 33% achieved CR [85]. CRS was observed in 84% of patients, and Grade 3 or above occurred in 5% of patients. ICANS occurred in 18% of patients, 3% of whom were Grade 3; No Grade 3 or above neurotoxicity occurred. Another trial (CRB-401, NCT02658929) also demonstrated the safety and efficiency of Abecma in heavily-pretreated R/R MM patients [87]. Of the 62 patients receiving infusion, the ORR was 76% (47 patients) and the CR was 39% (24 patients). Any Grade CRS was 76% (6% ≥ Grade 3, 4 patients). Any Grade ICANS was 44% (3% ≥ Grade 3, 2 patients).

Compared to other products, Abecma showed significant improvement in the incidence of Grade 3 or above AEs, however, there is a need to improve the CR..

Apart from bb2121 (Bluebird), MM can be treated using CAR-T cell therapies targeting BCMA through different mechanisms, such as LCAR-B38M (Legend), C-CAR088, and JCAR125. Among others.. LCAR-B38M is a structure-differentiated CAR-T cell therapy composed of 4-1BB and two single domain antibodies (VHH-VHH) that increase affinity to and target BCMA [88]. LEGEND-2 (NCT03090659) is an exploratory study of LCAR-B38M CAR-T cells, showing encouraging efficacy and controlled safety. Recently, researchers update long-term safety and efficacy data from a median follow-up of 4 years. In this trial, 74 patients with R/R MM received infusion [89]. Of them, AEs occurred in all patients, with 60.8% (45 patients) exhibiting Grade 3 or above AEs. CRS occurred in 91.9% of patients (68 patients); 9.5% (7 patients) were Grade 3 or above. Only one patient had a Grade 1 ICANS. The ORR and CR were 87.8 and 73.0% (54 patients), respectively. Compared to Abecma, LCAR-B38M has a higher ORR and CR, and its incidence of Grade 3 or above AEs is generally lower.

### Carvykti (Ciltacabtagene Autoleucel)

As the first Chinese CAR-T cell therapy (BCMA/VHH-VHH) to be approved by FDA on February 28, 2022, Carvykti is the second CAR-T cell therapy worldwide targeting BCMA. Carvykti is used after three or more prior lines of therapy, including immunomodulatory drugs, proteasome inhibitors as well as anti-CD38 antibodies, and is among the last treatment options for adults with progressed R/R MM [90]. Unlike previous CAR-T products, Carvykti contains two single domain VHH antibodies (VHH-VHH), which greatly enhance affinity to target cell surface BCMA.

The Phase 1b/2 clinical study, CARTITUDE-1 (NCT03548207) [90], was launched to assess the safety and clinical activities of Carvykti. In the most recent data [91], 97 patients had an ORR of 97.9%, a very good partial response (VGPR) of 94.9%, and a strict complete response (sCR) of 82.5%. The median times for first response, best response, and ≥ CR were 1.0, 2.6 and 2.9 months, respectively. The median duration of response (mDOR) was not reported [91]. Among the 97 patients admitted [92], ICANS occurred in 16 (16%) patients, while two patients had a Grade 3/4 event. From a raw-data perspective, Carvykti appears even better than Abecma.

### Relmacabtagene Autoleucel (Relma-cel, JWCAR029)

Relma-Cel (JWCAR029) (CD19/FMC-63) as a second line or above treatment is suitable for treating DLBCL patients. Transduced autologous CD4^+^and CD8^+^T cells express tEGFR and CD19-specific CARs in Relma-cel [93]. The CAR structure expressed by Relma-cel is comparable to that of another CAR-T cell product, Breyanzi (CD19/4-1BB/CD3-ζ). Breyanzi has a high response rate and is associated with low levels of toxicity in R/R LBCL.

Relma-cel optimized the Breyanzi manufacturing process to improve productivity as well as success rates and eliminate the need for separate CD4^+^and CD8^+^T-cell lines to provide doses with consistent product attributes [94]. In manufacturing, T cells are specifically enriched during blood component separation by employing CD4 and CD8 beads and selected by activation with CD3/CD28 beads. Moreover, in manufacturing process of Relma-cel, CD4^+^ and CD8^+^T cells are prepared separately and transfused in a non-fixed ratio [95].

In a Phase 2 clinical trial (NCT04089215) [93], Of the 59 treated patients receiving infusion, Any Grade CRS occurred in 47.5% of patients while CRS above Grade 3 was 5.1%. The incidence of ICANS at any grade was 20.3% while ICANS above Grade 3 was 5.1%. The ORR and CR were 75.9 and 51.7%, respectively. CRS and ICANS were controlled and all cases in the trial were resolved, except for one patient who died on Day 8 from sepsis with ongoing Grade 4 CRS, which was evaluated by the investigator as unrelated to CAR-T therapy. This trial demonstrates that Relma-cel has a comparable efficacy as Breyanzi in R/R LBCL with low levels of related toxicity and good therapeutic effects.

### Other CAR-T products

Autologous BG1805 (Anti-CLL1 CAR-T) was awarded the title of orphan drug by FDA for treating AML. CLL1 is highly expressed in AML cells and leukemia stem cells (LSCs) of children, but its expressions are markedly low in normal hematopoietic cells. In a Phase I clinical trial (chiCTR1900027684), 11 R/R AML children were treated, receiving BG1805. The median peak time of amplification in vivo was found to be 8 days, indicating rapid and efficient amplification of CAR-T cells [96]. Of these 11 children, the ORR was 81.9% and the overall disease control rate (DCR) was 90.9%. Among them 10 patients completely responded and CLL1^+^AML cancer cells were eliminated within 1 month [97]. Five patients achieved CR and minimal residual disease negative (MRD^**−**^), 3 patients achieved CR and MRD^+^, 1 patient achieved partial response (PR), while 1 patient showed stable disease (SD) with CLL1-negative AML cancer cells [97]. Grade 3/4 AEs were detected in 11 patients, but dose-dependent toxicity was not detected. CRS Grades 1 to 3 were observed, but there were no fatal events. CB-010 therapy is an editing-based allogeneic anti-CD19 CAR-T cell therapy established by CRISPR using the CRISPR heterozygous RNA-DNA (chRDNA) technology for gene editing. The gene editing technology is a chRDNA technology. Compared to CRISPR-Cas9, chRDNA has a high specificity and a low off-target editing level, allowing multiple gene editing and insertion. CB-010 therapy involves three gene editing aspects: (I) Site-specific knockdown of CD19 CAR into T cells; (II) Knockout of TRAC gene (constant region of T cell receptor α) to eliminate TCR and avoid GVHD; (III) Knockout of genes encoding PD-1. In its first clinical trial, Caribou Biosciences reported that 5 evaluable patients had ORR and CR of 100% of 80%, respectively. In this trial, after 3 months of treatment, CR was noted in the 4 patients, with a maximum duration of 6 months. Compared to prior CAR-T cell therapies, preliminary clinical data revealed a marked increase in ORR and CR and the therapeutic CB-010 dose was below and safer.

## CAR-T immunotherapy combination therapy

The application of CAR-T cell immunotherapy treating malignant B cells has shown some promise, however, its applications in solid malignant tumors are very limited. These limitations are attributed to several resistance mechanisms of CAR-T cells, including the absence of CAR-T cell persistence, target depletion, antigenic loss, and an immunosuppressed TME. CAR-T cell immunotherapy combined with targeted drugs has shown encouraging clinical outcomes. We discuss current trials of targeted therapy combined with CAR-T cell therapy to overcome resistance mechanisms, including the safety and efficiency of combination regimens. Some of the trials involving combination regimens are presented in Table 2 (See Supplementary Table 2, Additional file 2).

### PD-1/PD-L1

**Fig. 3:**
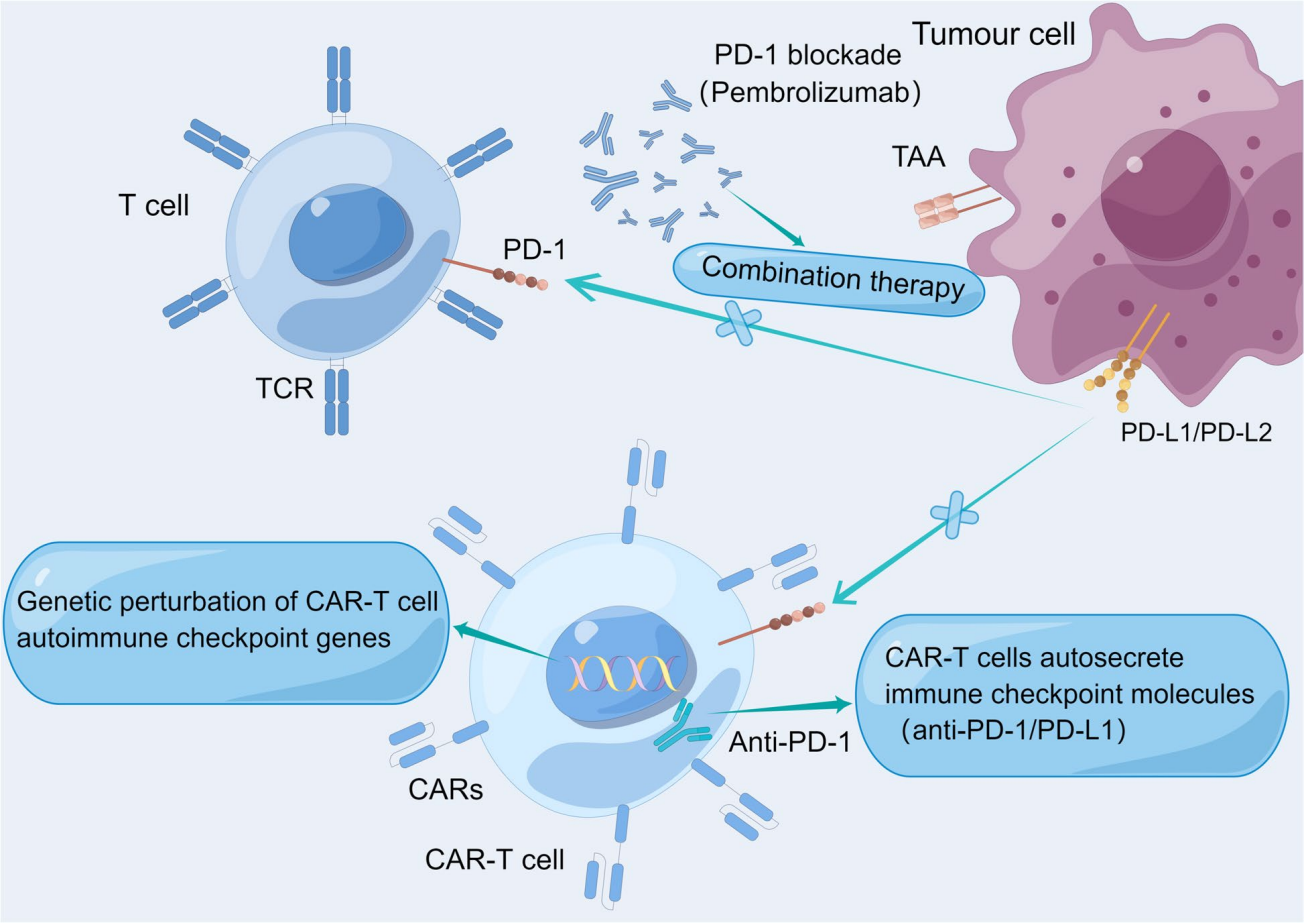
Application of immune checkpoint blocking technology in CAR-T cells. (I) Combination therapy, treated with pembrolizumab (anti-PD-1); (II) CAR-T cells self-secrete immune checkpoint molecules (III) Genetic perturbation of CAR-T cell autoimmune checkpoint genes. (By Figdraw)

The efficacies of CAR-T cell therapy combined with immune checkpoint inhibitors are currently being evaluated (Fig. 3). (I) Combination therapy. Pre-clinical studies have revealed that effector functions of CAR-T cells are usually exhausted, which limits their anti-tumor activities. The efficacy of combination therapy was documented in a case report of a DLBCL patient whose disease progression after receiving CD19-targeted CAR-T cell therapy (4-1BB/CD3-ζ) [98]. Pembrolizumab (anti-PD-1) was administered on Day 26. On Day 45, a significant tumor regression was observed, and the patient was allowed to return to work after 3 weeks; (II) CAR-T cells self-secrete immune checkpoint molecules. In preclinical mouse models, CAR-T cells secreted antibodies against immune checkpoints [99]. Additionally, such local secretion of antibodies against immune checkpoints may reduce systemic toxicity [100]; (III) Genetic perturbation of CAR-T cell autoimmune checkpoint genes, including CAR-T cell modification to make them resistant to PD-L1 [101] and knockout of CAR-T cell-encoding PD-1 gene using CRISPR-Cas9, thereby enhancing CAR-T cell functions in vitro and clearance of PD-L1 + tumor xenografts in vivo [102]. Moreover, the gene knockout method was clinically tolerable, and Cas9-edited CAR-T cells persisted in patients’ bodies of 9 months. Compared to 4-1BB CAR-T cells, the PD-1 signal may directly lead to dephosphorylation and inactivation of CD28 CAR-T cells, contributing to functional failure [103].

ZUMA-6 was a Phase 1/2 trial to investigate the safety and efficiency of Yescarta combined with atezolizumab (anti-PD-L1) for treating R/R DLBCL patients. After the Phase 1 trial, comparison of amplification levels of CAR-T cells (area of curves_D0–28_) revealed that the value in ZUMA-6 was twice the median value in ZUMA-1, while the median level was higher than that of ZUMA-1 after 28 days [104]. Data for Phase 1 Cohort 3 and Phase 2 patients (28 patients received infusion) revealed that optimal ORR is 75% while CR is 46%; 46% of patients were in a state of continuous response, however, median peak CAR-T cell levels for ZUMA-6 and ZUMA-1 as well as median values for CAR-T cell expansion as measured by area under the curve over the previous 28 days were similar. In summary, ZUMA-6, when compared to ZUMA-1, revealed a manageable safety profile for PD-L1 blocking when combined with atezolizumab following Yescarta infusions, low dose limiting toxicity (DLT) incidence, and without a significant increase in AEs incidences. However, efficacy and CAR-T cell levels after combination of Yescarta with atezolizumab were comparable to those in patients treated with Yescarta alone.

Although checkpoint blockade combined with CAR-T cell therapy is a promising immunotherapeutic option, the combination therapy might be insufficient in inducing T cell infiltrations and effector functions (ZUMA-6 results).

### Ibrutinib

Ibrutinib dysregulates the signaling pathway of the hematopoietic system by inhibiting BTK. (I) Ibrutinib directly affects T cells. In early clinical studies, ibrutinib has off-target effects and inhibits ITK (IL-2-inducible kinase) involved in TCR signal transduction. However, the inhibitory effect is limited to Th2 differentiated cells because Th1 and CD8^+^T cells have the RLK signal escape pathway [105]. Due to this selective inhibition of Th2 cells, CD4^+^T cells may differentiate into the Th1 subset and improve Th1 responses. Moreover, it can preferentially expand T-effector memory cell subsets, reduce PD-1 expressions on CD8^+^T cells and CTLA-4 on T cells, increase Th17 cells, and reduce the ratio of Treg cells to CD4^+^T cells [106–108]; (II) The ability to destroy the immunosuppressive TME effect. Many cells (nurse-like cells and mesenchymal stromal cells) presented in the chronic lymphocytic leukemia (CLL) microenvironment are necessary for survival, immune escape and proliferation of leukemia cells, while ibrutinib has the ability to mobilize CLL cells out of the secondary lymphoid organs [109, 110]. Ibrutinib suppresses CXCR4 expressions in leukemia cells to inhibit its downstream signal transduction [111]. When unable to return to the lymphoid tissue, leukemia cells are released into blood circulation. If targeted by CAR-T cells in blood circulation, then, the cytotoxic activity is increased and functional exhaustion of CAR-T cells is reduced. Besides, inhibitory effects of BTK can also disrupt cytokine-mediated translocation of Tumour Associated Macrophages (TAMs) into the TME [112], dysregulate NACHT, LRR, as well as NLRP3 in TAMs, and suppress tumorigenic IL-1β expressions [113]. Ibrutinib can also down-regulate CD200 (OX2) expressions in hematological malignancies [107].

In a Phase 1/2 study, Turtle et al. assessed CD19 CAR-T cell safety and feasibility in R/R CLL patients with ibrutinib treatment failure (all patients discontinued ibrutinib prior to lymphocyte clearance or leukocyte isolation) [114]. Four weeks after 24 patients with CLL received infusion, the ORR was 71% (17 patients). CRS and ICANS were noted in 20 and 8 patients, respectively. However, AEs were reversible in all but 1 patient. Based on this study, Gauthier et al. conducted another trial when concurrently administered with CD19 CAR-T cell therapy and compared the outcomes to those of a former cohort (without ibrutinib) [115]. Seventeen and 19 patients were treated in the ibrutinib and No-ibrutinib cohorts. The concurrent ibrutinib cohort exhibited low CRS severity compared to No-ibrutinib cohort (Grade ≥ 3 CRS: ibrutinib, 0%; No-ibrutinib, 26%; *p* = 0.05) and peak serum concentrations of CRS-related cytokines (IL-8, IL-15, MCP-1). Although milder illness may be responsible for low CRS severity, a high number of CAR-T cells were observed in the ibrutinib cohort. Ibrutinib-treated patients had a higher ORR compared to non-ibrutinib-treated patients (88% vs 56%, respectively, *p* = 0.06) [115].

Ibrutinib combined with CAR-T cell therapy is a reasonable approach for enhancing CAR-T cell functions. However, prospective clinical trials should be performed to evaluate the efficacy of the combination regimen and the low CRS associated with the use of ibrutinib.

### PI3K inhibitors

Inhibition of the PI3K-AkT-mTOR-c-myc signaling pathway can result in the production of numerous CAR-T cells with controllable cytotoxicity. (I) PI3K inhibitors have been shown to improve the efficacy of CAR-T cells. The PI3Kδ pathway is vital in functioning of B and T cells. Inhibition of this pathway has anti-tumor effects in non-Hodgkin lymphoma and CLL [105]. Therefore, it has been proposed that inhibition of this signaling pathway can suppress T-cell differentiation during the expansion of isolated T cells, thereby transforming the CAR-T phenotype into a less differentiated state that is less prone to rapid exhaustion. The PI3Kδ signal is directly activated downstream of TCR, and is important for T cell proliferation and functions. However, such a tonic signaling pathway reduces the proportion of immature and central memory T cells [105] by enhancing phenotypic differentiation of T cells into terminal effector T cells [116]. In vivo, treatment with the PI3Kδ inhibitor (idelalisib) enhances the proliferation of CAR-T cells, which are characterized by an expanded memory T cell compartment, longer survival, longer duration of tumor control [117], and increased anti-tumor activities against CD19^+^ [118], CD33^+^ [117], as well as mesothelin^+^ [119] target cells. In vitro amplification of CAR-T cells using idelalisib reduces the expressions of exhaustion markers (PD-1 and TIM-3), and increases the expressions of L-selectin [105, 119]. The PI3K/AkT axis is also inhibited by IL-15 [120] (reducing the activity of mTOR), PI3K and c-myc B cell adaptor [121], and AkT suppression [122]. (II) The PI3K inhibitor reduces the cytotoxic effects of CAR-T cell therapy. Duvelisib, a selective bis-PI3K-δ,γ, has been approved for treating R/R CLL and FL. Duvelisib suppressed the expressions of IL-6, a CRS replacement marker, reduced CRS, and maintained the functions of CAR-T19 (CD19-28BBζ) in vitro [123]. However, the reduction in efficiency of CAR-T19 was not statistically significant (~ 20%) [123].

CRB-402 is an ongoing multi-center Phase 1 clinical trial for treatment of R/R MM [124]. The bb21217 has the same CAR molecule as bb2121. However, during in vitro culture, the PI3K inhibitor (bb007) was shown to increase the number of memory-like T cells. The latest data shows that 46 patients have already received bb21217 and among them, 67% had CRS (2 patients ≥Grade 3) while 22% had ICANS (3 patients ≥Grade 3) [124]. Among them, 24 patients were confirmed to have responded, of whom 18% had ≥CR while 30% had VGPR [124]. Paired analysis of T cells from peripheral blood mononuclear cells and drug products from 44 patients revealed a significant increase in memory-like T cells (LEF1^+^, CD27^+^, CCR7^+^) and a reduction in highly differentiated and senescent CD57^+^cells after bb21217 treatment. In addition, it was postulated that the marked increase in early memory markers (LEF1^+^, CD27^+^, CCR7^+^) and low-expressions of depletion markers (EOMES^+^, GZMA^+^, and CD57^+^) could be due to higher CAR-T cell amplifications and longer reaction durations [124].

In conclusion, inhibition of the PI3K signaling pathway is a potential approach for generating numerous CAR-T cells, with a property of controllable cytotoxicity and early memory phenotype. However, studies should be performed to elucidate on the significance of PI3K inhibition in alleviating AEs.

## Advances in CAR-T therapy

It is challenging for CAR-T cell therapy to be efficacious against advanced CLL patients owning to “exhausted” T cells in the body after chemotherapy. Recently, University of Pennsylvania researchers used JQ1 as an experimental drug to inhibit the BET protein, thereby improving the functions of CAR-T cells [125]. Kong et al. proved that the BET protein can disrupt CAR expressions of T cells and key acetylated histone functions in CLL patients. Inhibition of the BET protein suppresses TET2 methylcytosine dioxygenase levels, and forced expression of the TET2 catalytic domain eliminates enhanced targeting of the BET protein in CAR-T cells.

Targeting two or more tumor-related molecules may prevent tumor cells from immune escape. Spiegel et al. evaluated 44 LBCL patients receiving standard CD19 CAR-T cell therapy [126]. Among them, 89% exhibited significantly expressed CD19 on the surface of pre-treated cancer cells. Approximately a half of these LBCL patients showed disease progression. Moreover, 9 of 15 (60%) were initially CD19-positive and had converted to CD19^−^ or low levels at the time of relapse, suggesting that these lymphoma cells may have escaped treatment. Spiegel et al. also found that patients with more than about 3000 CD19 molecules per cancer cell surface had a higher possibility of responding well, while those with fewer CD19 molecules were more likely to relapse after successful treatment.

Genome editing techniques are an significant push for the advancement of CAR-T therapy. CRISPR/Cas9 via Lentiviral delivery can be employed for the knockout of the endogenous TCR (eTCR) in tumour cells to enhance natural ability of T cells [127]. Furthermore, CRISPR/Cas9-mediated eTCR-knockout in CD19-CAR-T cells may avoid the risk of GVHD (alloreactivity), however, knockout of eTCR of CAR-T cells may reduce the longevity of the response [128]. CRISPR/Cas9 can also be utilized in combination with adeno-associated viral vectors for targeted integration into specific sites [129]. Additionally, genome editing techniques can be used for CAR-T cell manufacturing. In a recent study, researchers develop a good manufacturing practice-compatible process for nonviral CAR-T manufacturing [130]. Researchers develop single-stranded DNA homology-directed repair templates incorporating Cas9 target sequences (CTSs) with reduced toxicity, which enhance knock-in efficiencies (46–62%) and yields (> 1.5 × 10^9^ modified cells) by an additional roughly two-to threefold on average [130].

## Conclusions

Due to its safety and controllability, CAR-T immunotherapy has become a critical novel approach for treating some R/R malignant hematological diseases. However, its high costs, complex manufacturing processes and cytotoxicity are a challenge for its clinical applications. Various approaches for enhancing the manufacturing process while reducing cytotoxicity are being developed, including MASTER and chRDNA. Furthermore, its efficacy and duration are affected by many factors, including construction of the CAR molecule, the signal transduction mechanism, and TME among others. Currently, various strategies have been explored for CARs, including arming CAR-T to enhance its penetration into tumors and deleting immune checkpoint molecules via CRISPR/Cas9 to avoid depletion. Overall, studies should identify novel targets or develop the new CARs to achieve higher safety and efficacy effects.

Combinations of CAR-T cell therapy with PD-1/PD-L1, ibrutinib, PI3K inhibitors, and others have marked outcomes on blood cancer treatment. Combined therapy has been revealed to enhance the functions of CAR-T cells and delay their exhaustion, inhibit the TME, and reduce cytotoxic effects. Therefore, prospective clinical trials should be performed to prove the feasibility of these options. Finally, the application of genome editing techniques in CAR-T therapy is rapidly accelerating. Genome editing techniques (such as CRISPR/Cas9) can be utilized to avoid alloreactivity via knockout of endogenous TCR and provided a new CAR-T manufacturing with reduced toxicity and high efficiency.

## Supplementary Information

**Additional file 1.** Variations in efficacy and safety effects of different CAR-T products.

**Additional file 2.** Ongoing and completed clinical trials about combination therapies.

## Data Availability

Not applicable.

## Abbreviations

CAR-T: Chimeric antigen receptor T cells
CR: Complete response
ORR: Objective response rate
CRS: cytokine release syndrome
ICANS: Immune effector cell-associated neurotoxicity syndrome
CARs: CAR structures
VH: Variable heavy
VL: Variable light
scFv: Single-chain variable fragment
TAAs: Tumor-associated antigens
MHC: Major histocompatibility complex
VHH: Variable domain on a heavy chain
HcAbs: Variable domain of heavy chain-only antibodies
NF-κB: Nuclear factor-κB
TNF: Tumour necrosis factor
TRAFs: TNF receptor-associated factors
ITAMs: Immunoreceptor tyrosine-based activation motifs
ZAP70: Tyrosine kinase ζ-related protein of 70 kDa
IL: Interleukin
TRUCKs: T cells redirect for universal cytokine killings
TME: Tomour microenvironment
NFAT: Nuclear factor activated T cells:
IL-2Rβ: IL-2 receptor β-chain
TCR: T cell receptor
AML: Acute myeloid leukemia
MM: multiple myeloma
TSAs: Tumour specific antigens
BID: BH3-interaction domain death agonist
FADD: FAS-associated death domain protein
GVHD: Graft-versus-host disease
TALENS: Transcriptional activator-like effector nucleases
Treg: Regulatory T cells
MASTER: Multifunctional Alginate Scaffold for T Cell Engineering and Release
B-ALL: B-cell precursor acute lymphoblastic leukemia
R/R: Relapsed/refractory
LBCL: Large B-cell lymphoma
DLBCL: Diffuse large B-cell lymphoma
HGBCL: High grade B-cell lymphoma
FL: Follicular lymphoma
AEs: Adverse events
CARB: CAR-transduced B cells
BTK: Bruton's tyrosine kinase
PMBL: Primary mediastinal B-cell lymphoma
TFL: Transformed follicular lymphoma
MZL: Marginal zone lymphoma
MCL: Mantle cell lymphoma
tEGFR: Truncated epidermal growth factor receptors
Relma-cel: Relmacabtagene Autoleucel
VGPR: Very good partial response
sCR: Strict complete response
mDOR: Median duration of response
LSCs: Leukemia stem cells
DCR: Disease control rate
MRD: Minimal residual disease
PR: Partial response
SD: Stable disease
chRDNA: CRISPR heterozygous RNA-DNA
DLT: dose limiting toxicity
CLL: chronic lymphocytic leukemia
TAMs: Tumour Associated Macrophages
eTCR: endogenous TCR
CTSs: Cas9 target sequences
