# Supplementary material for: The journey of CAR-T therapy in hematological malignancies
Source: Mol Cancer. 2022 Oct 8;21:194. doi: 10.1186/s12943-022-01663-0 (PMC9547409; doi:10.1186/s12943-022-01663-0)
Supplement: Supplementary file 1 — Additional file 1. Variations in efficacy and safety effects of different CAR-T products. [file 12943_2022_1663_MOESM1_ESM.docx]

Table1. Variations in efficacy and safety effects of different CAR-T products

|  | Kymriah | | Yescarta | | | Tecartus | | Breyanzi | | Abecma | | Carvykti |
| --- | --- | --- | --- | --- | --- | --- | --- | --- | --- | --- | --- | --- |
| Target/Binder | CD19/FMC63 | | CD19/FMC63 | | | CD19/FMC63 | | CD19/FMC63 | | BCMA/C11D5.3 | | BCMA/VHH-VHH |
| Co-stimulatory domain | 4-1BB | | CD-28 | | | CD-28  CD3-ζ | | 4-1BB  CD3-ζ | | 4-1BB  CD3-ζ | | 4-1BB |
| Signaling domain | CD3-ζ | | CD3-ζ | | |  |  |  |  |  |  | CD3-ζ |
| Phase | Phase 2 | Phase 2 | Phase 2 | Phase 1/2 | Phase 3 | Phase 2 | Phase 2 | Phase 1 | Phase 3 | Phase 2 | Phase 1 | Phase 1b/2 |
| Dose level | 3.1X10^8^ cells | 0.6-6X10^8^ cells | 2X10^6^ cells | 2X10^6^ cells | 2X10^6^ cells | 2X10^6^ cells | 1X10^6^ cells | Level1: 50X10^6^ cells  Level2: 100X10^6^ cells  Level3: 150X10^6^ cells | 1X10^8^ cells | Level1: 150X10^6^ cells  Level2: 300X10^6^ cells  Level3: 450X10^6^ cells | Level1: 150X10^6^ cells  Level2: 300X10^6^ cells  Level3: 450X10^6^ cells  or 800X10^6^ cells | 0.75X10^6^ cells |
| Pathologies  included | DLBCL  TFL | FL | DLBCL  TFL  PMBCL | FL  MZL | LBCL | MCL | B-ALL | LBCL, MCL (after two lines therapy) | LBCL (after one line therapy) | MM | MM | MM |
| infused patients  (evaluable for analysis) | 111 (90) | 97 (94) | 108 (101) | 148 (104) | 170 (170) | 68 (60) | 55 (55) | 268 (255) | 90 (90) | 128 (128) | 62 (62) | 97 (97) |
| Conditioning  Chemotherapy | FLU 25 mg/m^2^  CY 250 mg/m^2^ | | FLU 30 mg/m^2^  CY 500 mg/m^2^ | | | FLU 30 mg/m^2^  CY 500 mg/m^2^ | FLI 25 mg/m^2^  CY 900 mg/m^2^ | FLU 30 mg/m^2^  CY 300 mg/m^2^ | | FLU 30 mg/m^2^  CY 300 mg/m^2^ | | FLU 30 mg/m^2^  CY 300 mg/m^2^ |
| ORR | 52% | 86% | 83% | 92% | 83% | 93% | NR | 73% | 86% | 73% | 76% | 97.9 |
| CR | 40% | 66% | 58% | 74% | 65% | 67% | 71% | 53% | 66% | 33% | 39% | 82.5% |
| CRS (3-4) | 22% | 0% | 11% | 7% | 6% | 15% | 24% | 2% | 1 patient | 5% | 6% | two patients |
| ICANS (3-4) | 12% | 1% | 32% | 19% | 21% | 31% | 25% | 10% | 4% | 3% | 3% | NR |
| Clinical Trial | JULIET  (NCT02445248) | ELARA  (NCT03568461) | ZUMA-1  (NCT02348216) | ZUMA-5  (NCT03105336) | ZUMA-7  (NCT03391466) | ZUMA-2  (NCT02601313) | ZUMA-3  (NCT02614066) | TRANSCEND NHL 001  (NCT02631044) | TRANSFORM  (NCT03575351) | KARMMA  (NCT03361748) | CRB-401  (NCT02658929) | CARTITUDE-1  (NCT03548207) |
| Reference | [1, 2] | [3] | [4] | [5] | [6] | [7] | [8] | [9] | [10] | [11] | [12] | [13, 14] |

Abbreviations: DLBCL, diffuse large B-cell lymphoma; TFL, transformed follicular lymphoma; PMBCL, primary mediastinal B-cell lymphoma; FL: follicular lymphoma; MZL: marginal zone lymphoma; LBCL: large B-cell lymphoma; MCL, mantle cell lymphoma; B-cell precursor acute lymphoblastic leukaemia: B-ALL; NR, not reported; MM, multiple myeloma; FLU, fludarabine; CY, cyclophosphamide; ORR, objective response rate; CR, complete response; CRS, cytokine release syndrome; ICANS, immune effector cell-associated neurotoxicity syndrome.

**References:**

1. Maude SL, Teachey DT, Rheingold SR, Shaw PA, Aplenc R, Barrett DM, et al. Sustained remissions with CD19-specific chimeric antigen receptor (CAR)-modified T cells in children with relapsed/refractory ALL. J CLIN ONCOL. 2016;34:3011.

2. Maude SL, Laetsch TW, Buechner J, Rives S, Boyer M, Bittencourt H, et al. Tisagenlecleucel in children and young adults with B-cell lymphoblastic leukemia. NEW ENGL J MED. 2018;378(5):439-48.

3. Schuster SJ, Dickinson MJ, Dreyling MH, Martínez J, Kolstad A, Butler JP, et al. Efficacy and safety of tisagenlecleucel (Tisa-cel) in adult patients (Pts) with relapsed/refractory follicular lymphoma (r/r FL): Primary analysis of the phase 2 Elara trial. J CLIN ONCOL. 2021;39:7508.

4. Neelapu SS, Locke FL, Bartlett NL, Lekakis LJ, Miklos DB, Jacobson CA, et al. Axicabtagene ciloleucel CAR T-cell therapy in refractory large B-cell lymphoma. NEW ENGL J MED. 2017;377(26):2531-44.

5. Jacobson CA, Chavez JC, Sehgal AR, William BM, Munoz J, Salles G, et al. Axicabtagene ciloleucel in relapsed or refractory indolent non-Hodgkin lymphoma (ZUMA-5): a single-arm, multicentre, phase 2 trial. The Lancet Oncology. 2022;23(1):91-103.

6. Locke FL, Miklos DB, Jacobson CA, Perales M, Kersten M, Oluwole OO, et al. Axicabtagene ciloleucel as second-line therapy for large B-cell lymphoma. NEW ENGL J MED. 2022;386(7):640-54.

7. Wang M, Munoz J, Goy A, Locke FL, Jacobson CA, Hill BT, et al. KTE-X19 CAR T-cell therapy in relapsed or refractory mantle-cell lymphoma. NEW ENGL J MED. 2020;382(14):1331-42.

8. Shah BD, Ghobadi A, Oluwole OO, Logan AC, Boissel N, Cassaday RD, et al. KTE-X19 for relapsed or refractory adult B-cell acute lymphoblastic leukaemia: phase 2 results of the single-arm, open-label, multicentre ZUMA-3 study. The Lancet. 2021;398(10299):491-502.

9. Ogasawara K, Dodds M, Mack T, Lymp J, Dell'Aringa J, Smith J. Population Cellular Kinetics of Lisocabtagene Maraleucel, an Autologous CD19-Directed Chimeric Antigen Receptor T-Cell Product, in Patients with Relapsed/Refractory Large B-Cell Lymphoma. CLIN PHARMACOKINET. 2021;60:1621-33.

10. Kamdar M, Solomon SR, Arnason JE, Johnston PB, Glass B, Bachanova V, et al. Lisocabtagene maraleucel (liso-cel), a CD19-directed chimeric antigen receptor (CAR) T cell therapy, versus standard of care (SOC) with salvage chemotherapy (CT) followed by autologous stem cell transplantation (ASCT) as second-line (2L) treatment in patients (Pts) with relapsed or refractory (R/R) large B-cell lymphoma (LBCL): results from the randomized phase 3 transform study. BLOOD. 2021;138:91.

11. Munshi NC, Anderson Jr LD, Shah N, Madduri D, Berdeja J, Lonial S, et al. Idecabtagene vicleucel in relapsed and refractory multiple myeloma. NEW ENGL J MED. 2021;384(8):705-16.

12. Lin Y, Raje NS, Berdeja JG, Siegel DS, Jagannath S, Madduri D, et al. Idecabtagene vicleucel (ide-cel, bb2121), a BCMA-directed CAR T cell therapy, in patients with relapsed and refractory multiple myeloma: updated results from phase 1 CRB-401 study. BLOOD. 2020;136:26-7.

13. Usmani SZ, Martin TG, Berdeja JG, Jakubowiak AJ, Agha ME, Cohen AD, et al. Phase 1b/2 study of ciltacabtagene autoleucel, a BCMA-directed CAR-T cell therapy, in patients with relapsed/refractory multiple myeloma (CARTITUDE-1): Two years post-LPI. J CLIN ONCOL. 2022;40:8028.

14. Cohen AD, Parekh S, Santomasso BD, Gállego Pérez-Larraya J, van de Donk NW, Arnulf B, et al. Incidence and management of CAR-T neurotoxicity in patients with multiple myeloma treated with ciltacabtagene autoleucel in CARTITUDE studies. BLOOD CANCER J. 2022;12(2):1-9.
