# Supplementary material for: The journey of CAR-T therapy in hematological malignancies
Source: Mol Cancer. 2022 Oct 8;21:194. doi: 10.1186/s12943-022-01663-0 (PMC9547409; doi:10.1186/s12943-022-01663-0)
Supplement: Supplementary file 2 — Additional file 2. Ongoing and completed clinical trials about combination therapies. [file 12943_2022_1663_MOESM2_ESM.docx]

Table2. Ongoing and completed clinical trials about combination therapies

| Trial number | Phase | Disease | Target | Product | Intervention | Response | Reference |
| --- | --- | --- | --- | --- | --- | --- | --- |
| ICI |  |  |  |  |  |  |  |
| NCT03310619 | 1/2 | NHL | CD19 | JCAR017 (4-1BB) | JCAR017 is combined with durvalumab | ongoing |  |
| NCT02926833  (ZUMA-6) | 1/2 | DLBCL | CD19 | Axi-cel | Axi-cel is combined with atezolizumab | Phase 1: ORR=90%; CR=60%  (12 evaluable patients)  CRS Grade 3/4=3 patients  ICANS Graed 3/4=6 patients  Phase 1 Cohort 3+Phase 2: ORR=75%; CR=46%  CRS Grade 3/4=4%  ICANS Graed3/4=29%  (28 patients were infused) | [1] |
| NCT02706405 | 1 | DLBCL, HGBCL, PMBCL | CD19 | JCAR014 (4-1BB-CD3ζ-tEGFR) | JCAR014 is combined with durvalumab | ORR=50%; CR=42%  (12 evaluable patients)  CRS (Grade 4)=38% (1 patients)  ICANS (Graed 3/4)=8% (0)  (13 evaluable patients) | [2] |
| NCT02650999 | 1/2 | DLBCL, MCL, FL | CD19 | Tisa-cel | pembrolizumab are employed in patients failing to respond or relapsing after Tisa-cel infusion | ORR=25% (3 of 12 patients; 1 CR; 2 PR) | [3] |
| NCT03630159 | 1 | DLBCL | CD19 | Tisa-cel | Tisa-cel is combined with pembrolizumab | 1 PR; 2 PD (8 evaluable patients) | [4] |
| NCT04003649 | 1 | GBM | IL13 Rα2 | IL13Rα2 CAR-T(4-1BB) | Arm 1: IL13Rα2+lpilimumab and nivolumab  2: IL13Rα2+nivolumab | ongoing |  |
| NCT01822652 | 1 | NB | GD2 | iC9-GD2 T cells (CD28-OX40 with inducible caspase9) | 2 doses of pembrolizumab on days-1 and +21 of CAR-T cell infusion | Best response was SD in 2 patients | [5] |
| Ibrutinib |  |  |  |  |  |  |  |
| NCT03331198  (TRANSCEND-CLL-004) | 1/2 | CLL/SLL | CD19 | JCAR017 (Liso-cel/4-1BB) | Phase 1 Arm 1: JCAR017 monotherapy  2: JCAR017+ibrutinib  Phase 2 Arm 1: JCAR017 (dose from the Phase 1 monotherapy arm) | Phase 1: ORR=82%; CR=45%  (22 evaluable patients)  CRS (Grade 3)=74% (9%)  ICANS (Graed 3/4)=39% (22%)  (23 evaluable patients)  Phase 2: ongoing | [6] |
| NCT02640209 | Pilot Trial | CLL/SLL | CD19 | CTL119 (humanized anti-CD19 scFv-4-1BB) | CTL119 are employed in CLL/SLL patients with SD or PR on ibrutinib | ORR=93%; CR=43%  CRS (Grade 3)=95% (16%)  (19 patients were infused) | [7] |
| NCT03960840 | 1 | CLL/SLL, DLBCL | CD19 | YTB323 (4-1BB) | Arm 1: Dose escalation and expansion YTB323 is combined with ibrutinib in CLL/SLL patients  2: Dose escalation and expansion of YTB323 monotherapy treat DLBCL  3: Dose escalation and expansion of YTB323 monotherapy treat adult ALL | CRS (Grade 4)=27% (7%)  ICANS (Graed 3)=47% (13%)  (15 evaluable patients)  DL1: ORR and CR=25% (4 evaluable patients)  DL2: ORR and CR=75% (8 evaluable patients)  DL3: ongoing | [8] |
| NCT01865617 | 1/2 | ALL, CLL, NHL | CD19 | JCAR014 (4-1BB-CD3ζ-tEGFR) | From at least 2 weeks previous to leukapheresis until 3 months post-JCAR014 infusion, JCAR014 with concurrent ibrutinib is to be administered | ORR=88%; CR=56%  CRS(Grade 3/4)=76% (0%) | [9] |
| PI3K inhibitors |  |  |  |  |  |  |  |
| NCT03274219 | 1 | MM | BCMA | bb21217 (the same CAR molecule as bb2121) | bb21217 (add the PI3K inhibitor bb007 during ex vivo culture) | CRS (Grade 3/5)=67% (1 G3, 2 G5)  ICANS (Graed 3/4)=22% (2 G3, 1 G4)  18% ≥CR and 30% with VGPR  (46 evaluable patients) | [10] |

Abbreviations: NHL, non-Hodgkin’s lymphoma; DLBCL, diffuse large B cell lymphoma; ORR, overall response rate; CR, complete response; CRS, cytokine release syndrome; ICANS, immune effector cell-associated neurotoxicity syndrome; Axi-cel, axicabtagene ciloleucel; HGBCL, high grade B cell lymphoma; PMBCL, primary mediastinal B cell lymphoma; MCL, mantle cell lymphoma; FL, follicular lymphoma; Tisa-cel, tisagenlecleucel; PR, partial response; PD, progressive disease; GBM, glioblastoma; NB, neuroblastoma; SD, stable disease; ALL, acute lymphocytic lymphoma; CLL, chronic lymphocytic lymphoma; Liso-cel, lisocabtagene maraleucel; SLL, small lymphocytic lymphoma; LBCL, large B cell lymphoma; MM, multiple myeloma; BCMA, B cell maturation agent; VGPR, very good partial response.

**References:**

1. Jacobson CA, Westin JR, Miklos DB, Herrera AF, Lee J, Seng J, et al. Abstract CT055: Phase 1/2 primary analysis of ZUMA-6: Axicabtagene ciloleucel (Axi-Cel) in combination With atezolizumab (Atezo) for the treatment of patients (Pts) with refractory diffuse large B cell lymphoma (DLBCL). CANCER RES. 2020;80:T55.

2. Hirayama AV, Gauthier J, Hay KA, Sheih A, Cherian S, Chen X, et al. Efficacy and toxicity of JCAR014 in combination with durvalumab for the treatment of patients with relapsed/refractory aggressive B-cell non-Hodgkin lymphoma. BLOOD. 2018;132(Supplement 1):1680.

3. Chong EA, Melenhorst JJ, Svoboda J, Nasta SD, Landsburg DJ, Mato AR, et al. Phase I/II study of pembrolizumab for progressive diffuse large B cell lymphoma after anti-CD19 directed chimeric antigen receptor modified T cell therapy. BLOOD. 2017;130:4121.

4. Jaeger U, Worel N, McGuirk JP, Riedell PA, Fleury I, Borchmann P, et al. Portia: a phase 1b study evaluating safety and efficacy of tisagenlecleucel and pembrolizumab in patients with relapsed/refractory diffuse large B-cell lymphoma. BLOOD. 2019;134:5325.

5. Heczey A, Louis CU. Advances in chimeric antigen receptor immunotherapy for neuroblastoma. DISCOV MED. 2013;16(90):287.

6. Siddiqi T, Soumerai JD, Dorritie KA, Stephens DM, Riedell PA, Arnason J, et al. Phase 1 TRANSCEND CLL 004 study of lisocabtagene maraleucel in patients with relapsed/refractory CLL or SLL. Blood, The Journal of the American Society of Hematology. 2022;139(12):1794-806.

7. Gill SI, Vides V, Frey NV, Metzger S, O'Brien M, Hexner E, et al. Prospective clinical trial of anti-CD19 CAR T cells in combination with ibrutinib for the treatment of chronic lymphocytic leukemia shows a high response rate. BLOOD. 2018;132:298.

8. Flinn IW, Jaeger U, Shah NN, Blaise D, Briones J, Shune L, et al. A First-in-Human Study of YTB323, a Novel, Autologous CD19-Directed CAR-T Cell Therapy Manufactured Using the Novel T-Charge TM platform, for the Treatment of Patients (Pts) with Relapsed/Refractory (r/r) Diffuse Large B-Cell Lymphoma (DLBCL). BLOOD. 2021;138:740.

9. Gauthier J, Hirayama AV, Hay KA, Li D, Lymp J, Sheih A, et al. Comparison of efficacy and toxicity of CD19-specific chimeric antigen receptor T-cells alone or in combination with ibrutinib for relapsed and/or refractory CLL. BLOOD. 2018;132(Supplement 1):299.

10. Alsina M, Shah N, Raje NS, Jagannath S, Madduri D, Kaufman JL, et al. Updated results from the phase I CRB-402 study of anti-Bcma CAR-T cell therapy bb21217 in patients with relapsed and refractory multiple myeloma: correlation of expansion and duration of response with T cell phenotypes. BLOOD. 2020;136:25-6.
